# Life-Space Activities Are Associated with the Prognosis of Older Adults with Cardiovascular Disease

**DOI:** 10.3390/jcdd9100323

**Published:** 2022-09-24

**Authors:** Kakeru Hashimoto, Akihiro Hirashiki, Koharu Oya, Junpei Sugioka, Shunya Tanioku, Kenji Sato, Ikue Ueda, Naoki Itoh, Manabu Kokubo, Atsuya Shimizu, Hitoshi Kagaya, Izumi Kondo

**Affiliations:** 1 Department of Rehabilitation, National Center for Geriatrics and Gerontology, Obu 474-8511, Japan; 2 Department of Cardiology, National Center for Geriatrics and Gerontology, Obu 474-8511, Japan

**Keywords:** life-space assessment, life-space activities, cardiovascular events, heart failure, cardiovascular disease, older adult patient

## Abstract

Life-space activities are a measure of daily activity level. Here, we examined the association between life-space activities and prognosis in 129 cardiovascular diseases (CVD) patients 65 years of age or older (average age, 79.2 ± 7.6 years; mean left ventricular ejection fraction, 56.7 ± 13.2%) who had been admitted to our hospital for worsening CVD. Subjects were followed, and the primary endpoints were cardiovascular hospitalization and cardiovascular death. Receiver operating characteristic analysis produced a cutoff value for life-space assessment (LSA) score for increased risk of cardiovascular hospitalization for two years of 53.0 points (sensitivity, 55.9%; specificity, 82.1%). Kaplan–Meier analysis using this cutoff value revealed that the rates of cardiovascular hospitalization and cardiovascular death were significantly higher in subjects with an LSA score below the cutoff than in those with a score above the cutoff (both *p* < 0.001). Cox proportional analysis revealed that low LSA score was independently associated with cardiovascular hospitalization (HR, 2.540; 95% CI, 1.135–5.680; *p* = 0.023) and cardiovascular death (HR, 15.223; 95% CI, 1.689–137.180; *p* = 0.015), even after adjusting for age, sex, left ventricular ejection fraction, and log-transformed brain natriuretic peptide level. Thus, life-space activities are associated with prognosis in older adults with CVD.

## 1. Introduction

The prevalence of cardiovascular disease (CVD) increases with age [1]. Older adults with CVD often have multiple comorbidities [2], including reduced exercise capacity, decreased physical and cognitive functions, and depression. In addition, they frequently have problems specific to older adults, such as frailty [3] and sarcopenia [4]. These comorbidities increase the mortality and readmission rates of CVD patients [2].

It is also common for older adults to experience a decline in life-space activities [5]. Life-space activities are a concept used to assess patterns of functional mobility over time [6]. Life-space activities are measured by using the Life-Space Assessment (LSA) tool that provides a score indicating the degree of use of the immediate life space and the wider environment by a patient; the higher the LSA score, the shorter the sedentary time and more physical activity a patient undertakes [7].

Decreased life-space activities lead to decreased physical and cognitive functions and an increased risk of falls [8]. In addition, lower life-space activities contribute to increased mortality and readmission rates for community-dwelling older adults [8] and patients with the chronic obstructive pulmonary disease [9] or heart failure [10]. As classic prognostic indicators in patients with CVD, especially heart failure, brain natriuretic peptide (BNP) and left ventricular ejection fraction (LVEF) are known [2,4]. However, neither the association between LSA and prognosis nor the usefulness of LSA compared with conventional markers in older adults with CVD has yet been reported.

Here we sought to examine whether LSA is associated with prognosis in older adult patients with CVD.

## 2. Materials and Methods

### 2.1. Study Population

This was a longitudinal study conducted on patients who had been admitted for worsening CVD to the cardiology department of the National Center for Geriatrics and Gerontology (Obu, Japan) between August 2016 and August 2021. All subjects were at least 65 years old and were able to perform a cardiopulmonary exercise test (CPX); undergo laboratory measurements, echocardiography, and a physical function evaluation; and complete questionnaires; these examinations were conducted prior to discharge after the patients had been medically stabilized. After discharge, all patients were prospectively followed up for the occurrence of cardiovascular events. Noncardiac death was excluded.

The inclusion criteria were structural heart disease involving coronary artery disease (having experienced angina pectoris or myocardial infarction or with a history of having undergone a revascularization procedure), symptomatic heart failure (non-ischemic cardiomyopathy, tachycardia, bradycardia, valvular, or hypertension), or other (aortic disease, peripheral artery disease, thoracic aortic aneurysm, or abdominal aortic aneurysm). Non-ischemic cardiomyopathies were defined as ventricular myocardial abnormalities in the absence of coronary artery disease or valvular, pericardial, or congenital heart disease [11]. Tachycardia and bradycardia included atrial, supraventricular, and ventricular arrhythmias; sick sinus syndrome; and atrioventricular block in the absence of structural heart disease. Valvular heart disease was diagnosed based on hemodynamic or echocardiographic findings or a history of valvular or congenital cardiac surgery, according to the American College of Cardiology and American Heart Association Guideline for the Management of Patients with Valvular Heart Disease [12]. Hypertension was defined as systolic blood pressure ≥140 mmHg, diastolic blood pressure ≥90 mmHg, or a history of treatment for hypertension. Worsening heart failure was defined as a clinical syndrome comprising symptoms and/or signs due to structural and/or functional cardiac abnormality and accompanied by elevated natriuretic peptide levels and/or objective evidence of pulmonary or systemic congestion [13].

The exclusion criteria were severe respiratory dysfunction (i.e., patients receiving long-term oxygen therapy due to respiratory disease), liver dysfunction (Child-Pugh class C), stroke, renal dysfunction (glomerular filtration rate stage G5), malignant tumors with a prognosis of less than one year, difficulty walking 10 m even with a walking aid, a Mini-Mental State Examination (MMSE) score of fewer than 18 points, and living in a nursing care facility before hospital admission for CVD.

The study protocol complied with the Declaration of Helsinki and written informed consent was obtained from each subject. The ethics review board of the National Center for Geriatric and Gerontology approved the study (approval no. 1272).

### 2.2. Clinical Characteristics

Frailty was defined according to the revised Japanese version of the Cardiovascular Health Study (J-CHS) criteria [14]. The J-CHS assesses five components: weight loss, physical activity, tiredness, muscle weakness, and gait speed. Frailty was defined as the presence of signs or symptoms associated with at least three of the five components, prefrailty as the presence of signs or symptoms associated with one or two components, and robust as the absence of signs or symptoms associated with any of the components.

### 2.3. LSA

The Japanese version of the LSA was used (Figure S1, see Supplementary Materials). The LSA is a validated tool that measures community mobility based on the frequency of travel to various locations, or “life-space levels”, and the need for assistance to reach those levels in the 4 weeks before assessment. The LSA takes 10 to 15 min to administer by telephone, which was the mode of administration in the E-Coach trial. Specifically, the LSA asks: “During the past4 weeks, have you: been to other rooms in your home besides the room where you sleep (Level 1); been to an area outside your home such as your porch, deck or patio, the hallway of an apartment building, or garage (Level 2); been to places in your neighborhood other than your own yard or apartment building (Level 3); been to places outside your neighborhood but within your town (Level 4); and been to places outside your town? (Level 5)” For each location, participants were also asked how many days during the week they reached that location and whether they needed help from an assistive device or another person to get there. A composite score was calculated based on life-space level, degree of independence in achieving each level, and frequency of attaining each level. Life-space composite scores ranged from 0 to 120, with higher scores representing greater community mobility. An example of how the LSA is scored can be found elsewhere [15].

### 2.4. Measurements

Peak oxygen uptake (peak VO_2_), Short Physical Performance Battery (SPPB) score, Geriatric Depression Scale (GDS), and MMSE were assessed prior to discharge after the patients had been medically stabilized.

#### 2.4.1. CPX

Each patient underwent CPX on a cycle ergometer at a progressively increasing work rate to maximal tolerance. The test protocol was conducted in accordance with the recommendations of the American Thoracic Society and the American College of Chest Physicians [16]. Gas-exchange data were obtained breath-by-breath, and peak VO_2_ was determined as the highest value of oxygen uptake achieved during the test.

#### 2.4.2. SPPB

The SPPB evaluates lower limb function [17]. It has three components: balance test (closed leg standing, semi-tandem standing, tandem standing), walking time, and standing from a seated position. Its reliability, validity, and feasibility in older adults have been reported [18]. The maximum score is 12 points, and the higher the score, the better the patient’s physical function.

#### 2.4.3. GDS

The GDS is a 15-item indicator of depression that is used in Japan [19]. The maximum score is 15 points, and a higher score indicates a patient with more severe depression. A score of 10 or more indicates the presence of depression.

#### 2.4.4. MMSE

The MMSE measures cognitive function by using a point system that ranges from 0 to 30 points [20]. The lower the score, the lower the patient’s cognitive function.

### 2.5. Cardiovascular Events

Cardiovascular events were defined as cardiovascular hospitalization or cardiovascular death. Cardiovascular hospitalization was defined as hospitalization for worsening heart failure, acute coronary syndrome, or coronary revascularization. Cardiovascular death was defined as sudden cardiac death due to worsening heart failure or other cardiovascular death (i.e., cerebrovascular events, acute myocardial infarction, aortic vascular disease, or peripheral arterial disease).

### 2.6. Statistical Analysis

Continuous variables are expressed as mean ± standard deviation (mean ± SD). Categorical data are reported as the percentage of all subjects. Receiver operating characteristic (ROC) analysis was used to determine the area under the curve, sensitivity, specificity, and cutoff value for LSA score for increased risk of cardiovascular hospitalization within two years. The cutoff value was determined by using the maximum value of the Youden Index. Subjects were classified into two groups according to the determined LSA score cutoff value; subjects with an LSA score less than the cutoff value were placed in the low LSA group, and those with an LSA score greater than or equal to the cutoff value were placed in the high LSA group. For differences in the measurements between the two groups, normally distributed variables were compared by Student’s *t*-test, non-normally distributed variables by the Mann–Whitney U test, and categorical variables were assessed by the Chi-squared analysis. Moreover, the relationships between LSA and the two endpoint cardiovascular events were examined by survival time analysis (Kaplan–Meier method) and Cox proportional hazards analysis. In the Kaplan–Meier analysis, the log-rank test was used to examine the differences in event-free survival between the groups. In the Cox proportional hazards analysis, cardiovascular events were used as the dependent variables, and age, sex, and LVEF, and log-transformed BNP (log BNP), which are established prognostic markers for worsening heart failure [2,4], were used as the explanatory variables. The statistical analyses were performed using SPSS software version 27 (SPSS Inc., Chicago, IL, USA). A value of *p* < 0.05 was considered statistically significant.

## 3. Results

### 3.1. Baseline Clinical Characteristics

The baseline clinical characteristics of the 129 patients enrolled in the study are shown in Table 1. The average age was 79.2 ± 7.6 years; 46.5% of the subjects were men, the mean brain natriuretic peptide (BNP) level was 163.8 ± 157.2 pg/mL, mean left ventricular ejection fraction (LVEF) was 56.7% ± 13.2%, and mean LSA score was 74.1 ± 32.5 points. During follow-up, 27.9% of the subjects experienced cardiovascular hospitalization, and 8.5% experienced cardiovascular death.

### 3.2. ROC Analysis

**Figure 1:**
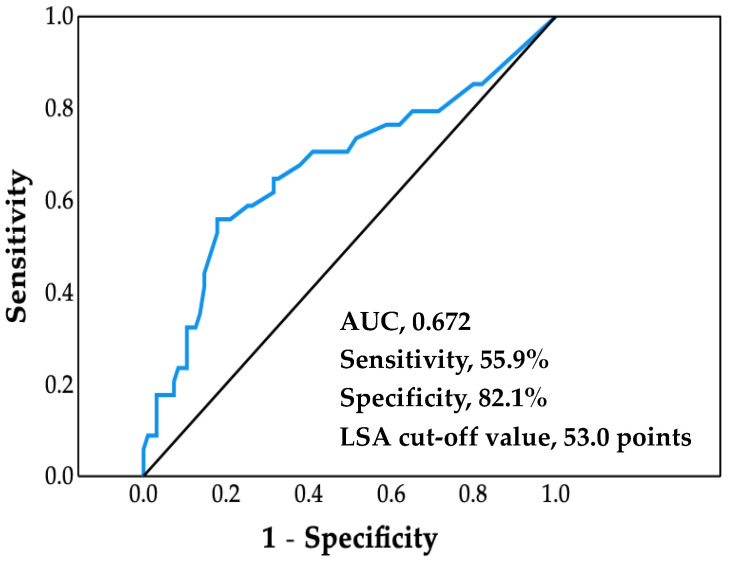
Receiver operating characteristic curve. Life-space assessment (LSA) cutoff value was determined by using the maximum value of the Youden Index. AUC, area under the curve.

The ROC analysis afforded a cutoff value of LSA for increased risk of cardiovascular hospitalization of 53.0 points (area under the curve, 0.672; sensitivity, 55.9%; specificity, 82.1%; *p* = 0.003) (Figure 1).

### 3.3. Comparison of Baseline Clinical Characteristics between LSA Groups

The mean LSA score in the low LSA group (32.3 ± 15.5 points) was significantly lower than that in the high LSA group (90.3 ± 20.8 points) (*p* < 0.001) (Table 2). Mean age, BNP level, and GDS score were significantly higher, and peak VO_2_, mean SPPB score and mean MMSE score were significantly lower in the low LSA group than in the high LSA group (*p* < 0.05). LVEF, underlying diseases, and medications were not significantly comparable between the two groups. The number of cardiovascular hospitalizations in the low LSA group (19 [52.8%]) was significantly higher than that in the high LSA group (17 [17.0%]) (*p* < 0.001). The number of cardiovascular deaths in the low LSA group (8 [22.2%]) was also significantly higher than that in the high LSA group (3 [3.0%]) (*p* = 0.012).

### 3.4. Survival Time Analysis

**Figure 2:**
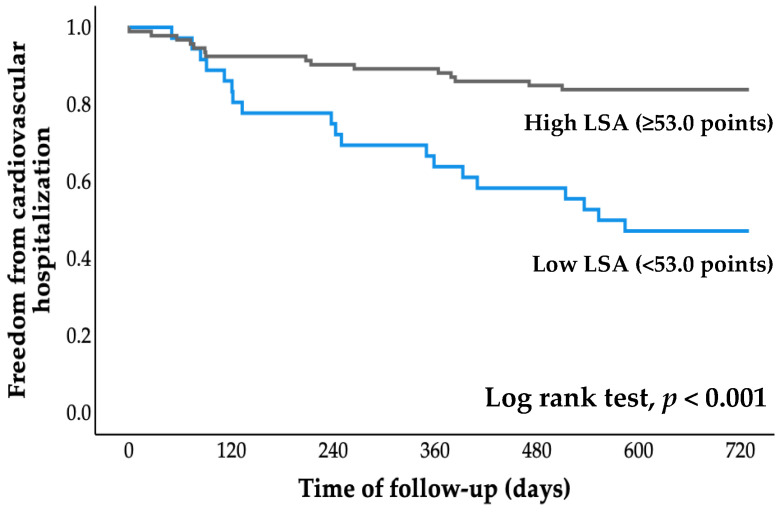
Kaplan–Meier curve for cardiovascular hospitalization. LSA, life-space assessment.

**Figure 3:**
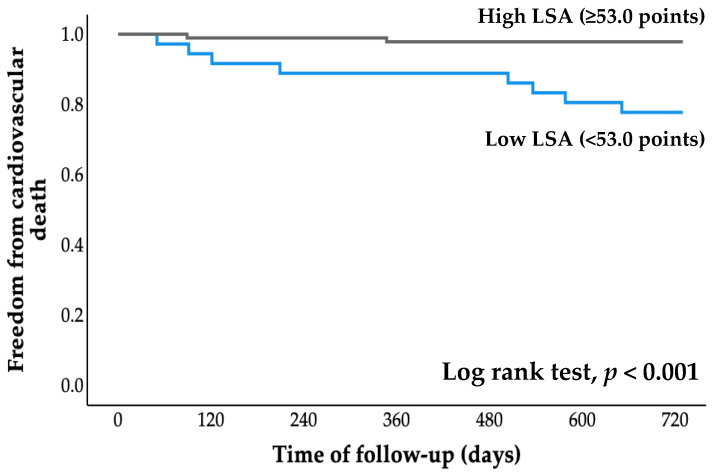
Kaplan–Meier curve for cardiovascular death. LSA, life-space assessment.

All patients were followed for an average of 2.7 years (range, 0.8 to 4.1 years), starting at the time of entry and ending with a cardiovascular event or the most recent evaluation of survivors. The cumulative probability of event-free survival was calculated using Kaplan–Meier analysis (Figure 2 and Figure 3). The probabilities of cardiovascular hospitalization and cardiovascular mortality in the low LSA group were significantly higher than those in the high LSA group (both *p* < 0.001, log-rank test).

### 3.5. Cox Proportional Hazards Analysis

Table 3 and Table 4 show the hazard ratio for cardiovascular hospitalization and cardiovascular death (model 1, model 2 and model 3). After adjusting for age and sex, low LSA score was extracted as an independent factor for cardiovascular hospitalization (hazard ratio, 2.420; 95% CI, 1.156–5.069; *p* = 0.019) and cardiovascular death (hazard ratio, 4.791; 95% CI, 1.119–20.502, *p* = 0.035) (Table 3 and Table 4, model 2). Furthermore, after adjusting for age, sex, LVEF and log BNP, LSA score was again extracted as an independent factor for cardiovascular hospitalization (hazard ratio, 2.515; 95% CI, 1.154–5.482; *p* = 0.020) and cardiovascular death (hazard ratio, 8.192; 95% CI, 1.507–44.541; *p* = 0.015) (Table 3 and Table 4, model 3).

## 4. Discussion

There are three main findings from the present study: (1) ROC analysis revealed a cutoff LSA score for increased risk of cardiovascular hospitalization of 53.0 points; (2) Kaplan–Meier analysis revealed that cardiovascular hospitalization and cardiovascular mortality rates were significantly higher in the low LSA group than in the high LSA group; and (3) Cox proportional hazards analysis revealed that low LSA score was independently associated with cardiovascular hospitalization and cardiovascular death, even after adjusting for age, sex, LVEF, and log BNP level. Thus, this study is the first to show that life-space activities are associated with both cardiovascular hospitalization and cardiovascular death in older adults with CVD.

The literature contains several LSA cutoff values for community-dwelling individuals. For example, cutoff values of 52.3 points and 56 points have been reported to predict a decline both in activities of daily living and instrumental activities of daily living over a two-year [21] and one-year period in older adults [22], respectively. A previous study with five-year cognitive decline as a primary endpoint has reported that subjects with LSA ≥61 points showed slower cognitive decline than those with LSA <61 points [23]. In the present study, the cutoff value for increased risk of cardiovascular hospitalization was 53 points.

Life-space activities are reported to be associated with healthcare utilization, admission to nursing homes, and mortality in community-dwelling older adults with osteoporosis [6]. Decreased life-space activities are also reported to be associated with an increased risk of readmission in patients with the chronic obstructive pulmonary disease [9] or heart failure [10]. In the present study, the LSA score was a significant predictor of cardiovascular hospitalization and cardiovascular death, even when adjusted for age, sex, LVEF and log BNP. In our population, 87.7% of the subjects were admitted to our hospital due to worsening heart failure (Table 1). In our previous cross-sectional study, we found that in addition to an assessment of clinical cardiac function, an assessment of motor function and social factors might also be important to understand the complete context of life-space activity in older adults with CVD [24]. The present study was a medium-term longitudinal study to elucidate the relationship between LSA and prognosis in a similar population, and the Cox proportional hazards analysis revealed that life-space activities are independently associated with cardiovascular events after adjusting for age and disease severity.

Coronary risk factors, cardiac function, BNP, exercise tolerance, physical inactivity, physical function, depression, and frailty are factors known to affect the prognosis of CVD, including heart failure [2,25]. In older adults, physical function, irrespective of the presence or absence of complications, is more related to prognosis than cardiac function and BNP level [26]. The present study further demonstrates the importance of factors other than cardiac function and BNP in the prognosis of older adults with CVD. LSA is reported to be associated not only with BNP but also with physical function, physical activity, depression, cognitive function, and social factors [24,27]. LSA is associated with multiple prognostic factors for CVD. However, these causal relationships could not be examined in the present study. Therefore, in the future, it will be necessary to clarify these causal relationships and consider whether LSA is directly or indirectly related to prognosis.

The present study has several limitations. It was a single-center study that included a small number of subjects, it used a short follow-up period, and it considered few cases of cardiovascular events. In addition, only four factors (age, sex, LVEF and log BNP) were adjusted for; therefore, the effects of CVD severity and pathophysiology (e.g., preserved vs. reduced ejection fraction) were not investigated.

## 5. Conclusions

A low LSA score is associated with a poor prognosis in older adults with CVD. In particular, LSA was an independent predictor of both cardiovascular hospitalization and cardiovascular death, even after adjusting for age, sex, LVEF, and log BNP. Thus, a combined evaluation of LSA and cardiovascular index might provide important information for improving clinical management and prognosis in older adults with CVD. In the future, further large-scale studies are needed to examine these associations to confirm. Finally, the association between prognosis and changeable LSA in older adults with CVD remains unclear and deserves further investigation.

## Figures and Tables

**Table 1:** Baseline clinical characteristics (n = 129).

Age (years)	79.2 ± 7.6
Sex (male, n (%))	60 (46.5)
Body mass index (kg/m^2^)	22.3 ± 3.9
**Underlying diseases**	
Worsening heart failure (n, %)	113 (87.7)
Non-ischemic cardiomyopathy (n, %)	8 (6.2)
Ischemic heart disease (n, %)	31 (24.0)
Tachycardia-induced (n, %)	34 (26.4)
Bradycardia (n, %)	10 (7.8)
Valve (n, %)	14 (10.9)
Hypertension (n, %)	10 (7.8)
Other (n, %)	6 (4.6)
Post PCI or CABG (n, %)	16 (12.3)
**Medication**	
Diuretic (n, %)	61 (47.3)
Tolvaptan (n, %)	17 (13.2)
ACE-I/ARB (n, %)	55 (42.6)
β blocker (n, %)	59 (45.7)
Spironolactone (n, %)	26 (20.2)
Anticoagulant (n, %)	55 (42.6)
Antiplatelet (n, %)	59 (45.7)
**Laboratory data**	
BNP (pg/mL)	163.8 ± 157.2
Hemoglobin (mg/dL)	12.5 ± 1.8
Total protein (g/dL)	6.8 ± 0.6
Albumin (d/dL)	3.8 ± 0.5
Total cholesterol (mg/dL)	172.8 ± 34.6
eGFR (mL/min/1.73 m^2^)	56.0 ± 18.5
**Echocardiography**	
LVEF (%)	56.7 ± 13.2
E/e′	15.6 ± 6.6
**Frailty**	
J-CHS (robust/prefrailty/frailty) (%)	4.4/54.4/41.2
**Other status**	
LSA (points)	74.1 ± 32.5
Peak VO_2_ (mL/min/kg)	13.1 ± 3.4
SPPB (points)	9.4 ± 2.8
GDS (points)	4.0 ± 3.2
MMSE (points)	27.0 ± 2.9
**Cardiovascular events**	
Cardiovascular hospitalization (n, %)	36 (27.9)
Cardiovascular death (n, %)	11 (8.5)

Mean ± standard deviation. ACE-I/ARB, angiotensin-converting enzyme inhibitor/angiotensin II receptor blocker; BNP, brain natriuretic peptide; CABG, coronary artery bypass graft; E/e′, the ratio of transmitral Doppler early filling velocity to tissue Doppler early diastolic mitral annular velocity; eGFR, estimated glomerular filtration rate; GDS, Geriatric Depression Scale; J-CHS, the Japanese version of the Cardiovascular Health Study criteria; LSA, life-space assessment; LVEF, left ventricular ejection fraction; MMSE, Mini-Mental State Examination; PCI, percutaneous coronary intervention; peak VO_2_, peak oxygen uptake; SPPB, Short Physical Performance Battery.

**Table 2:** Comparison of baseline clinical characteristics between the low and high LSA groups.

	Low LSA Group (n = 36)	High LSA Group (n = 93)	*p* Value
LSA (points)	32.3 ± 15.5	90.3 ± 20.8	<0.001
Age (years)	84.4 ± 6.4	77.4 ± 6.7	<0.001
Male (n, %)	20 (56)	40 (43)	0.166
**Underlying diseases**			
Worsening heart failure (n, %)	35 (94.4)	65 (85.0)	0.142
Non-ischemic cardiomyopathy (n, %)	3 (8.3)	5 (5.4)	
Ischemic heart disease (n, %)	6 (16.7)	25 (26.9)	
Tachycardia-induced (n, %)	7 (19.3)	27 (29.0)	
Bradycardia (n, %)	6 (16.7)	4 (4.3)	
Valve (n, %)	6 (16.7)	8 (8.6)	
Hypertension (n, %)	4 (11.1)	6 (6.5)	
Other (n, %)	2 (5.6)	4 (4.3)	
Post PCI or CABG (n, %)	2 (5.6)	14 (15.0)	
**Medication**			
Diuretic (n, %)	22 (61.1)	39 (41.9)	0.051
Tolvaptan (n, %)	6 (16.7)	11 (11.8)	0.466
ACE-I/ARB (n, %)	14 (38.9)	41 (44.1)	0.592
β blocker (n, %)	12 (33.3)	47 (50.5)	0.079
Spironolactone (n, %)	8 (22.2)	18 (19.4)	0.716
Anticoagulant (n, %)	14 (38.9)	41 (44.1)	0.592
Antiplatelet (n, %)	12 (33.3)	47 (50.5)	0.079
BNP (pg/mL)	199.3 ± 139.7	139.2 ± 142.1	0.035
LVEF (%)	58.1 ± 13.4	56.9 ± 12.5	0.644
Peak VO_2_ (mL/min/kg)	11.3 ± 3.0	13.9 ± 3.4	0.001
SPPB (points)	6.8 ± 3.2	10.3 ± 2.0	<0.001
GDS (points)	6.1 ± 3.1	3.3 ± 2.9	<0.001
MMSE (points)	25.3 ± 3.2	27.6 ± 2.6	<0.001
Cardiovascular hospitalization (n, %)	19 (52.8)	17 (17.0)	<0.001
Cardiovascular death (n, %)	8 (22.2)	3 (3.0)	0.012

Mean ± standard deviation. Abbreviations are as in Table 1. Low LSA group (LSA < 53.0 points); high LSA group (LSA ≥ 53.0 points). Groups were compared by unpaired *t*-test, Mann–Whitney U test, or chi-squared test.

**Table 3:** Cox proportional hazards analysis (cardiovascular hospitalization).

	Model 1			Model 2			Model 3		
	HR	95% CI	*p* Value	HR	95% CI	*p* Value	HR	95% CI	*p* Value
LSA									
≥53 points	Reference			Reference			Reference		
<53 points	3.717	1.928–7.164	<0.001	2.420	1.156–5.069	0.019	2.515	1.154–5.482	0.020
Age				1.064	1.013–1.117	0.013	1.039	0.989–1.092	0.126
Sex				0.901	0.461–1.760	0.760	0.921	0.467–1.816	0.812
LVEF							0.989	0.996–1.013	0.367
Log BNP							2.639	1.129–6.166	0.025

HR, hazard ratio; Log BNP, log-transformed brain natriuretic peptide. Other abbreviations are as in Table 1. Model 1: LSA; Model 2: model 1 added to age and sex; Model 3: model 2 added to LVEF and log BNP.

**Table 4:** Cox proportional hazards analysis (cardiovascular death).

	Model 1			Model 2			Model 3		
	HR	95% CI	*p* Value	HR	95% CI	*p* Value	HR	95% CI	*p* Value
LSA									
≥53 points	Reference			Reference			Reference		
<53 points	11.27	2.392–53.081	0.002	4.791	1.119–20.502	0.035	8.192	1.507–44.541	0.015
Age				1.090	0.995–1.194	0.065	1.052	0.948–1.167	0.337
Sex				1.129	0.327–3.902	0.847	1.315	0.353–4.902	0.683
LVEF							0.989	0.946–1.034	0.626
Log BNP							2.006	0.379–10.613	0.413

Abbreviations are as in Table 1 and Table 3. Model 1; LSA; Model 2; model 1 added to age and sex; model 3; model 2 added to LVEF and log BNP.

## Data Availability

Not applicable.

## Supplementary Materials

The following supporting information can be downloaded at: https://www.mdpi.com/article/10.3390/jcdd9100323/s1: LSA evaluation form.
