# Supplementary material for: Life-Space Activities Are Associated with the Prognosis of Older Adults with Cardiovascular Disease
Source: J Cardiovasc Dev Dis. 2022 Sep 24;9(10):323. doi: 10.3390/jcdd9100323 (PMC9604436; doi:10.3390/jcdd9100323)
Supplement: Supplementary file 1 [file jcdd-09-00323-s001.zip › jcdd-1912308-supplementary.pdf]

These questions refer to your activities just within the past month			
**Life-space level**		**Frequency**	**Independence**
During the past four weeks, have you been to…		How often did you get there ?	Did you use aids or equipment ? Did you need help from another person ?
**Level 1**<br>Other rooms of your home bedsides the room where you sleep ?	1. Yes<br>2. No	1. Less then 1/week<br>2. 1-3 times/week<br>3. 4-6 times/week<br>4. Daily	1. Personal assistance<br>2. Equipment only<br>3. No equipment or personal assistance
**Level 2**<br>An area outside your home, such as your. porch, deck or patio, hallway (of an apartment building) or garage, In your own yard or driveway ?	1. Yes<br>2. No	1. Less then 1/week<br>2. 1-3 times/week<br>3. 4-6 times/week<br>4. Daily	1. Personal assistance<br>2. Equipment only<br>3. No equipment or personal assistance
**Level 3**<br>Places in your neighborhood, other than your own yard or apartment building ?	1. Yes<br>2. No	1. Less then 1/week<br>2. 1-3 times/week<br>3. 4-6 times/week<br>4. Daily	1. Personal assistance<br>2. Equipment only<br>3. No equipment or personal assistance
**Level 4**<br>Places outside your neighborhood but within your town ?	1. Yes<br>2. No	1. Less then 1/week<br>2. 1-3 times/week<br>3. 4-6 times/week<br>4. Daily	1. Personal assistance<br>2. Equipment only<br>3. No equipment or personal assistance
**Level 5**<br>Places outside your town ?	1. Yes<br>2. No	1. Less then 1/week<br>2. 1-3 times/week<br>3. 4-6 times/week<br>4. Daily	1. Personal assistance<br>2. Equipment only<br>3. No equipment or personal assistance
